# Eco-Friendly Electrophoretic Deposition of Fluorescent Nanocomposite Films in an Aqueous Dispersion of Hydrophilized Core/Shell CuInS_2_/ZnS Quantum Dots for Optoelectronic Applications

**DOI:** 10.1021/acsami.3c17264

**Published:** 2024-02-05

**Authors:** Asshu Morimoto, Yoshiki Iso, Tetsuhiko Isobe

**Affiliations:** Department of Applied Chemistry, Faculty of Science and Technology, Keio University, 3-14-1 Hiyoshi, Kohoku-ku, Yokohama 223-8522, Japan

**Keywords:** fluorescent quantum dot, nanophosphor, flexible nanocomposite film, hydrophilization, aqueous electrophoretic deposition, low toxicity and environmental load, photoluminescence, white LED

## Abstract

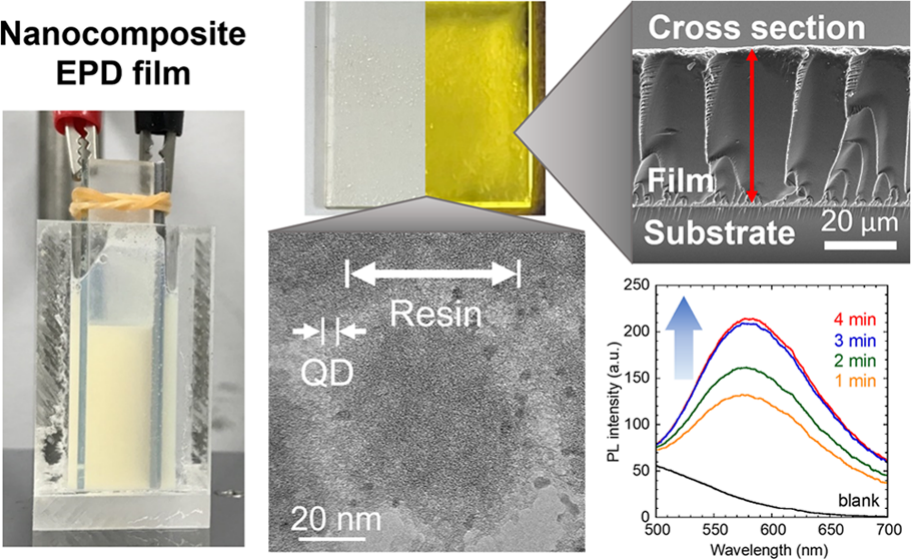

Low-toxic and efficient fluorescent core–shell CuInS_2_/ZnS (CIS/ZnS) quantum dots (QDs) are good candidates for optoelectronic device applications. They are synthesized in a hydrophobic environment, while large amounts of organic solvents used in the preparation of fluorescent films have significant problems on environmental load and human health. CIS/ZnS QDs hydrophilized by adsorbing 3-mercaptopropionic acid on their surfaces can be used in the aqueous film fabrication process. In this work, the aqueous electrophoretic deposition (EPD) of the hydrophilized QDs with silicone-modified acrylic resin nanoparticles was performed to fabricate fluorescent nanocomposite films. The hydrophilized QDs and resin nanoparticles were simultaneously dispersed in basic aqueous solutions due to electrostatic repulsion resulting from their negatively charged surfaces. Transparent films were obtained on a transparent conductive substrate at the anode side by the EPD. They showed yellow fluorescence of the QDs. The thickness increased with increasing the deposition time; however, hemispherical holes attributed to oxygen gas generated by water electrolysis were observed at the longer time. The electron microscopy revealed that the films were densely and homogeneously deposited. The QDs were dispersed around the resin nanoparticles without aggregation. The fluorescence (FL) quantum yield was 43%. The optical absorption peak and FL intensity of the QDs increased accompanied by the film growth. The nanocomposite film showed good heat resistance at 80–120 °C for 5 h; therefore, the prepared films have feasibility in white light-emitting diode (LED) applications. A lightening device structured with the obtained EPD film placed on a blue LED successfully emitted white light. In addition, the flexibility of the nanocomposite film was demonstrated. The aqueous EPD method would be one of the suitable methods for the industrial production of fluorescent QD films. This technique can be applied to other hydrophilic fluorescent QDs with charged surfaces. Realization of various fluorescent QD films would expand the application possibilities.

## 1 Introduction

Fluorescent quantum dots (QDs), which are inorganic semiconductor nanocrystals, are phosphor materials with properties such as controllable band gap by quantum confinement effect, higher durability than organic materials, and high fluorescence quantum yield (FLQY).^1−4^ Due to their great fluorescence (FL) properties, QDs are widely applied to optoelectronic devices such as QDs displays, lasers, and FL imaging in medicine.^5−10^ However, Cd- and Pb-based QDs such as CdSe and CsPbBr_3_ are restricted by the RoHS Directive due to their toxicity; therefore, alternative materials with lower toxicity are highly desired.^11−13^ CuInS_2_ (CIS) QDs are attractive nanophosphors that exhibit visible FL without Cd and Pb. They show lower self-absorption due to their larger Stokes shift caused by the Cu defect-related radiative recombination after interband excitation.^14−16^ Furthermore, high FLQY is achieved by ZnS shelling to passivate surface defects of the CIS core causing the nonradiative recombination.^17^ Core/shell CIS/ZnS QDs exhibiting up to 97% FLQY have been reported.^18^ They are attractive for various applications such as white light-emitting diodes (LEDs), solar cells, and bioimaging due to their low toxicity and excellent visible FL properties.^19−23^

When fluorescent films using QDs are applied to optoelectronic devices, nanocomposites are frequently prepared by dispersing QDs in a resin matrix to protect the QDs, avoid concentration quenching, and provide flexibility. However, the dispersibility of nanoparticles is sensitive to changes in affinity for the surrounding dispersant or matrix, which easily leads to aggregation. In our previous study, hydrophobic CIS/ZnS and CuGaS_2_/ZnS QDs were dispersed in toluene dissolving ethylene-vinyl acetate copolymer to prepare fluorescent nanocomposite films by a simple casting method; however, the clear dispersions become opaque films due to the significant QD aggregation.^22,24^ Excess concentration of QDs occurred during the drying process. Aggregation of QDs leads to a decrease in FLQY due to concentration quenching and a red-shift in emission wavelength due to increased self-absorption. Translucency of the films is reduced by enhanced light scattering due to aggregation.

We have focused on electrophoretic deposition (EPD) to fabricate nanocomposite films of nanophosphors.^25−27^ EPD is a method of depositing a film on an electrode by electrophoresis of particles with a charged surface by applying voltage between electrodes in a dispersion.^28−30^ Uniform films are obtained from a dilute colloidal solution in a short time and at low cost.^31^ Several kinds of particles with a charged surface of the same sign can be dispersed in a dispersant; they are simultaneously deposited on a conductive substrate by the EPD method. The film thickness can be controlled by adjusting the deposition time and applied voltage. Moreover, aggregation is hard to occur because the dispersed particles are directly deposited without a drying process.^32,33^ Efficient fluorescent QDs are synthesized in liquid phases with a hydrophobic environment. EPD can be performed in hydrophobic solvents. Fulari et al. prepared fluorescent films of CsPbBr_3_ nanocrystals modified with polyvinylpyrrolidone by EPD in toluene for optoelectronic device applications.^34^ However, such a hydrophobic EPD uses a large amount of organic solvents. In order to reduce the environmental load and avoid health hazards, water is preferred to use instead of organic solvents. Hydrophobic QDs can be dispersed in water through hydrophilization techniques by surface modification; therefore, we expect that an aqueous EPD of hydrophilized QDs would be achieved. To the best of our knowledge, there have been only a few reports on aqueous EPD using hydrophilic fluorescent nanoparticles. In the previous study, we deposited silicone-modified acrylic resin and YVO_4_:Bi^3+^, Eu^3+^ nanoparticles simultaneously by aqueous EPD to fabricate transparent fluorescent nanocomposite films.^27^ Uniform films of micrometer thickness were obtained from the two types of nanoparticles with negatively charged surfaces. The use of a resin also made them flexible. Such fluorescent films would be applicable in flexible optoelectronic devices.

Hydrophobic QDs are hydrophilized by exchanging hydrophobic surface ligands with hydrophilic ligands.^13^ Choi et al. prepared efficient fluorescent CIS/ZnS QDs with both ligands of 1-dodecanethiol (DDT) and decylamine, which is an easily exchangeable ligand. The QDs maintained a high FLQY of 60% after hydrophilization by ligand exchange to 11-mercapto-1-undecanol.^35^ In our previous study, hydrophobic surface ligands with an alkyl chain were replaced by 3-mercaptopropionic acid (MPA) to achieve hydrophilicity for CIS/ZnS QDs and InP/ZnS QDs.^36−38^ The hydrophilized CIS/ZnS QDs using MPA were more dispersible in basic water than those using 11-mercapto-1-undecanol.^36^ The carboxyl group of MPA is negatively charged by deprotonation under basic conditions, which causes electrostatic repulsion between particles.

In this study, transparent fluorescent nanocomposite films were prepared by aqueous EPD from MPA-modified CIS/ZnS QDs and a silicone-modified acrylic resin. Herein, both nanomaterials are codispersed in basic water due to their negatively charged surfaces. Silicone-modified acrylic resin has high transparency in the near-ultraviolet (UV), visible, and near-infrared regions and exhibits good light and heat resistance. The use of a resin matrix is necessary to suppress concentration quenching of QDs to maintain higher FLQY.^36^ Furthermore, this resin provides flexibility for the nanocomposite films.^39,40^ The negatively charged QDs and resin nanoparticles are expected to be simultaneously deposited on the anode under voltage application. Nanocomposite films were fabricated on ITO-coated glass substrates by aqueous EPD with varying deposition times. Their morphologies, FL properties, and durability were evaluated.

## 2 Experimental Section

### 2.1 Materials

Zinc(II) acetate dihydrate (99.9%), copper(I) iodide (99.999%), sodium hydroxide (97.0%), 15% aqueous tetramethylammonium hydroxide (TMAOH) solution were purchased from FUJIFILM Wako Pure Chemical Corporation. Indium(III) acetate (99.99%), 15–20 wt % aqueous tetramethylammonium silicate (TMAS) solution were purchased from Sigma-Aldrich. Oleic acid (OA; >85.0%), DDT (>95.0%), 1-octadecene (ODE; >90.0%), and MPA (>98%) were purchased from the Tokyo Chemical Industry. Aqueous emulsion of silicone-modified acrylic resin (35 wt %) was received from DAI NIPPON TORYO. Ethanol (99.5%) and hexane (95.0%) were purchased from Kanto Chemical Industries. Ethanol and hexane were dehydrated over molecular sieves (3A 1/16, Kanto Chemical) prior to use.

### 2.2 Synthesis of CIS/ZnS QDs

A mixture of zinc(II) acetate dihydrate (4.0 mmol), OA (1.5 mL), DDT (1 mL), and ODE (4 mL) was heated at 190 °C for 5 min. The mixture was bubbled with Ar for 30 min to obtain a ZnS shell stock solution. Two stock solutions were prepared for the double shelling process described below. DDT (5.0 mL) was placed in a four-neck flask and bubbled with Ar for 30 min, followed by the addition of copper(I) iodide (0.125 mmol) and indium(III) acetate (0.500 mmol). The ZnS shell stock solution was placed in a funnel connected to the four-necked flask. The reaction system was degassed at 100 °C for 30 min under vigorous stirring and then purged with Ar. The temperature was increased to 230 °C and maintained for 5 min to nucleate and grow the core CIS QDs. The ZnS shell stock solution was then dropped into the resulting colloidal solution at a rate of 1.0 mL min^–1^. The temperature was raised to 240 °C and maintained for 50 min to grow the CIS/ZnS core/shell QDs. Another ZnS shell stock solution in a syringe was then added to the CIS/ZnS QDs dispersion at the same rate. The mixture was aged for 60 min for further shell growth. After rapid cooling to room temperature with water, the as-synthesized QD dispersion was added with toluene (7.5 mL) and ethanol (15 mL), and then centrifuged at ∼16 000 g (12 000 rpm using a rotor of 10 cm in radius) for 10 min. The obtained precipitate was redispersed in toluene (5 mL) under ultrasonication. After addition of ethanol (15 mL), the precipitate was again collected by centrifugation at ∼16 000 g for 10 min. This cycle of redispersion and centrifugation was performed twice for purification. A powder sample of the QDs was obtained by drying the resulting precipitate in a vacuum overnight.

### 2.3 Hydrophilization of the CIS/ZnS QDs through a Ligand Exchange Method

The ligand exchange for the hydrophobic CIS/ZnS QDs was performed by an in situ method. MPA (6 mL) was injected into the dispersion of as-synthesized QDs at 240 °C after 50 min of aging for the second shell growth. This mixture was aged for 15 min and then rapidly cooled to room temperature with water. The resulting QD dispersion was dispersed in ethanol (10 mL) under the ultrasonication. After the addition of hexane (20 mL), a precipitate was collected by centrifugation at ∼11 000 g (8000 rpm using a rotor of 10 cm radius) for 10 min. This cycle of redispersion and centrifugation was repeated three times for purification. Vacuum drying of the obtained precipitate was performed overnight to obtain a powder sample of the hydrophilized QDs.

### 2.4 Fabrication of Nanocomposite Films by EPD

**Figure 1:**
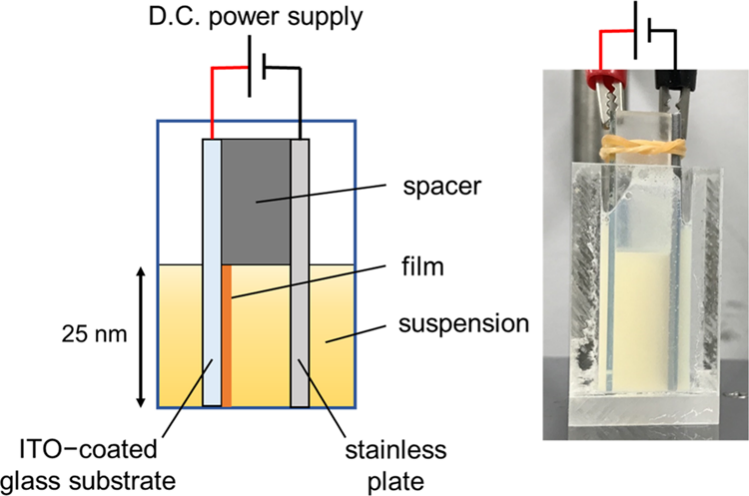
Schematic illustration and photograph of the EPD system using an ITO-coated glass substrate. It should be noted that the dispersion was not transparent because the resin suspension used was opaque.

To obtain the dispersion used in EPD at pH 10.0, the pH value of the 15 wt % aqueous TMAOH solution was adjusted to 13.2 by dilution with ultrapure water. The hydrophilized QDs (120 mg) were dispersed in this solution (4.0 mL) under ultrasonication. The 35 wt % aqueous emulsion of silicone-modified acrylic resin (8.0 mL; pH 7.5) was added and ultrasonicated. The final pH value of the resulting dispersion was 10.0. The weight ratio of QDs/resin in the dispersion was calculated as 3.4:96.6. The resulting suspension was used in the following EPD process. As shown in Figure 1, an ITO-coated glass substrate (25 mm × 50 mm × 1 mm, 30 Ω sq^–1^) and a stainless-steel plate (25 mm × 50 mm × 1 mm, SUS-304) were used as the cathode and anode, respectively. The distance between the two electrodes was maintained at 10 mm by using an insulating spacer. They were vertically immersed in the prepared dispersion for EPD to a depth of 25 mm. The EPD apparatus was the same as that used in our previous works.^25−27^ A QD composite film was deposited on a 25 mm × 25 mm area of the glass substrate by applying a constant voltage of 4.0 V for 1–5 min. Here, 4.0 V was the minimum voltage required for the film deposition from our preliminary experiment. For aqueous EPDs, lower voltages are desirable to suppress the electrolysis of water. After EPD, the films were rinsed with ultrapure water to remove excess dispersion, followed by drying at 120 °C for 15 min to obtain the film samples.

### 2.5 Characterization

X-ray diffraction (XRD) profiles were measured on an X-ray diffractometer (Rigaku, MiniFlex600) by using monochromatic Cu Kα radiation. Fourier-transform infrared (FT-IR) absorption spectra were measured on an FT-IR spectrometer (JASCO, FT/IR-4200). Electron microscopy was performed with a transmission electron microscope (TEM; FEI, Tecnai G2) and a scanning electron microscope (SEM; FEI, Inspect F50). QD dispersions were dropped onto a microgrid (Oken Shoji, COL-M15) and vacuum-dried for TEM observation. The microgrid was attached to the ITO-coated glass substrate with carbon tape to perform TEM observation on an EPD film. The deposition time was 5 s to prepare the thin film. FL microscopy was performed by using an FL microscope (Nikon, EclipseE600) equipped with a digital camera. The filter block set was composed of an excitation filter (330–380 nm), an FL filter (580LP), and a dichroic mirror reflecting 505 nm light. X-ray FL (XRF) spectra were measured by using an X-ray FL spectrometer (Bruker, M4 TORNADO) to determine elemental compositions. UV–visible (UV–vis) absorption spectra of QD dispersions were measured with an optical absorption spectrometer (JASCO, V-750). The Tauc plot was calculated according to the eq 1 to determine the *E*_g_ of the QDs.^41^
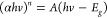
1where α is the absorbance, *h* is the Planck constant, ν is the frequency, and *A* is a constant. Herein, *n* was 1/2 because the optical absorption of CIS is attributed to direct interband transition.^42^ UV–vis absorption and transmission spectra of EPD films were measured on an optical absorption spectrometer (JASCO, V-750), equipped with an integrating sphere (JASCO, ISV-922). FL excitation and emission spectra of QDs dispersions were measured by using an FL spectrometer (JASCO, FP-8500). The obtained spectra were calibrated by using a calibrated detector (JASCO, SID-844). FL spectra of EPD films were measured by a FL spectrometer (JASCO, FP-6500), equipped with an integrating sphere (JASCO, ISF-513) (see also Figure S1). The spectral response was calibrated using an ethylene glycol solution of rhodamine B (5.5 g L^–1^) and a secondary standard light source (JASCO, ESC-333). Absolute FLQYs for QD dispersions and an EPD film were measured by using a quantum efficiency measurement system (Otsuka Electronics, QE-2000-311C). In thermal stability tests, transmission and FL spectra of the obtained films were measured before and after heating at 80, 120, and 160 °C for 5 h. These measurements were performed after the mixture was cooled to room temperature to avoid temperature quenching. Thermogravimetry and differential thermal analysis (TG-DTA; Rigaku, Thermo Plus TG 8120) was performed in an air flow of 300 mL min^–1^ at a heating rate of 10 K min^–1^ with Al_2_O_3_ as a reference. The emission spectrum of an assembled white LED device was measured by an LED evaluation system (Hamamatsu Photonics, C9920-22).

## 3 Results and Discussion

### 3.1 Characterization of Hydrophobic and Hydrophilized CIS/ZnS QDs

Crystal structures of both QDs were evaluated by the XRD method. Judging from the profiles shown in Figure S2, peaks of tetragonal chalcopyrite-type CIS and sphalerite-type ZnS were observed for them. Their positions and widths did not change, revealing that the crystal structure was maintained even after the hydrophilization process. Figure S3 shows TEM images of the prepared QDs. Nanoparticles smaller than ∼3 nm were observed, regardless of the hydrophilization process. The lattice fringe spacing on the hydrophobic QDs was 3.1 Å, corresponding to the (112) plane of CIS or the (111) plane of ZnS. In the hydrophilized QDs, the lattice fringe spacing was 3.2 Å, indicating that there was no change in the crystal structure. As exhibited in the TEM image of Figure S4, the average size of the silicone-modified acrylic resin nanoparticles was 54.6 ± 9.9 nm, which was ∼20 times larger than that of the QDs.

**Figure 2:**
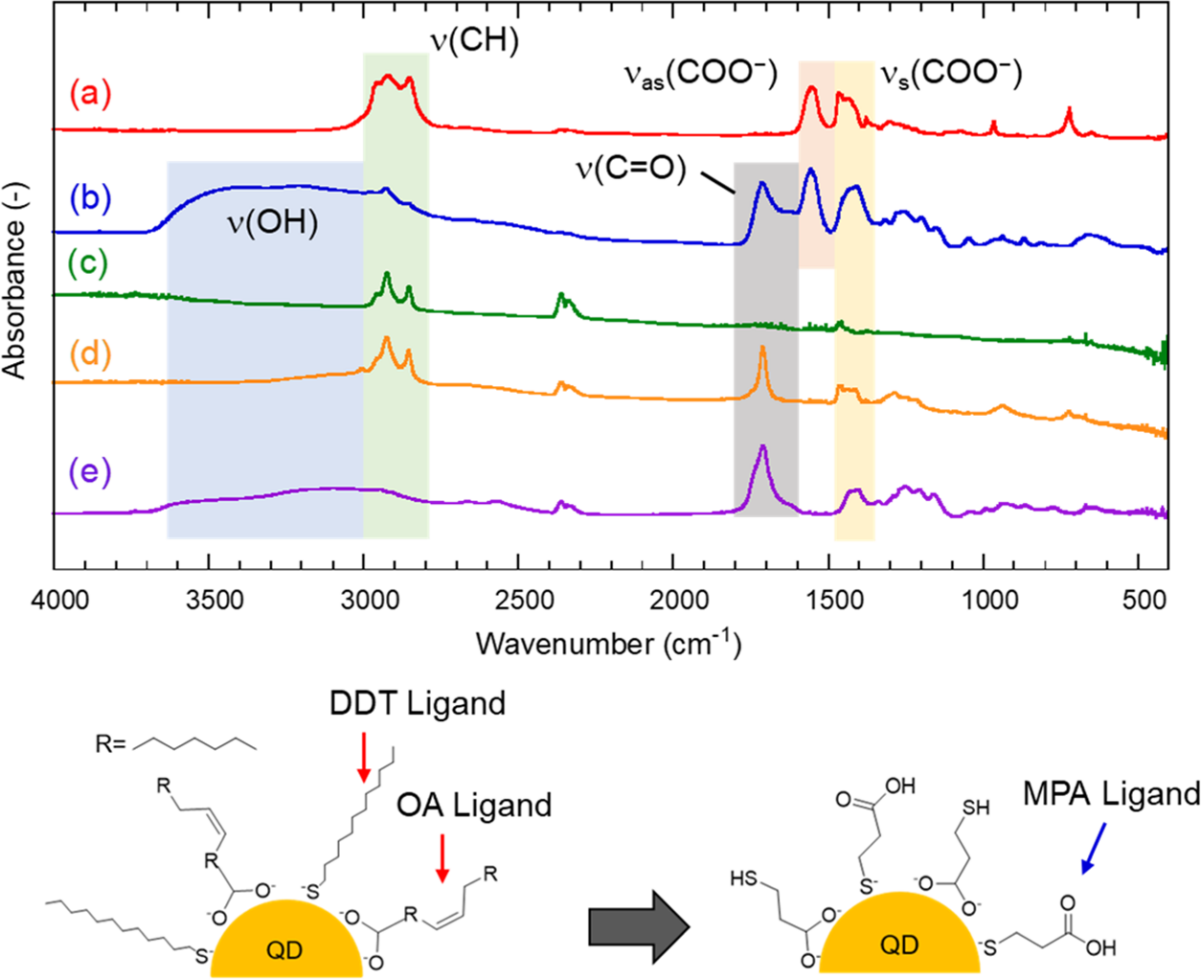
FT-IR spectra of (a) hydrophobic QDs, (b) hydrophilized QDs, (c) DDT, (d) OA, and (e) MPA. Schematic illustration of the ligand exchange is also shown.

The ligand exchange on the QD surface for the hydrophilization was confirmed by the FT-IR spectra shown in Figure 2. The hydrophobic QDs showed strong C–H stretching vibration {ν(CH)} peaks at around 2900 cm^–1^. They are attributed to the alkyl chain groups of DDT and OA adsorbed on the hydrophobic QD surface. On the other hand, their peak intensities decreased after the hydrophilization process. This indicates that DDT and OA with long alkyl groups were replaced by MPA, while they might partially remain for the hydrophilized QDs.^36^ The absorption peaks of symmetric and asymmetric stretching vibrations {ν_s_(COO^–^) and ν_as_(COO^–^) } of the COO^–^ groups were observed at ∼1400 and ∼1560 cm^–1^ before and after hydrophilization, respectively. These can be attributed to OA and MPA. The absorption peak that appeared at ∼1700 cm^–1^ observed for the hydrophilized QDs is assigned to C=O stretching vibration {ν(C=O)} of the COOH group in MPA. The broad peak of OH at ∼3500 cm^–1^ is attributed to stretching vibration {ν(OH)} of MPA and H_2_O adsorbed on the hydrophilized QDs. The residual H_2_O could be derived from ethanol used for the QD collection process or moisture. These FT-IR results indicate that the hydrophobic QDs were successfully hydrophilized through ligand exchange of DDT and OA for MPA.

### 3.2 Characterization of Aqueous QD Dispersions

To deprotonate the COOH group of MPA on the QDs for stable dispersion using sufficient electrostatic repulsion, the aqueous dispersant must be in basic conditions. The dispersibility of hydrophilized QDs in basic aqueous solutions of TMAS, TMAOH, and NaOH was compared. The aqueous QD dispersions are displayed in Figure S5. The QDs were dispersed at each appropriate pH of the basic condition. When the deprotonation of the COOH group in MPA was not sufficient at lower pH, the QDs exhibited strong aggregation due to insufficient negative surface charges. It should be noted that the QD dispersion of NaOH at pH 13.0 was turbid, indicating that the excessively basic environment reduced the dispersibility, possibly caused by degradation of the QD surface. FLQYs were measured for the clear dispersions and are summarized in Table 1. Their values decreased with an increasing pH value, indicating that the QDs were degraded by the excessively basic conditions. The maximum FLQY was 15% in the TMAS and TMAOH solutions. TMAOH solution was chosen as the dispersant for the EPD process to avoid possible silicate precipitation from TMAS on an electrode. To suppress the degradation of QDs in the excessively basic environment, the dispersion used in the EPD process should be prepared at a lower pH value. Interestingly, aggregation of QDs occurred in TMAOH solution at pH 11.5 as shown in Figure S5; however, no sedimentation was observed in the prepared dispersion used in EPD at pH 10.0 as displayed in Figure 1.

**Table 1:** FLQY of CIS/ZnS QDs in Each Basic Aqueous Solution

dispersant	pH	FLQY (%)
TMAS aq.	10.5	15
	11.0	13
	12.0	13
TMAOH aq.	11.5	15
	12.0	14
	13.9	9
NaOH aq.	11.5	13
	12.0	13
	13.0	10

**Figure 3:**
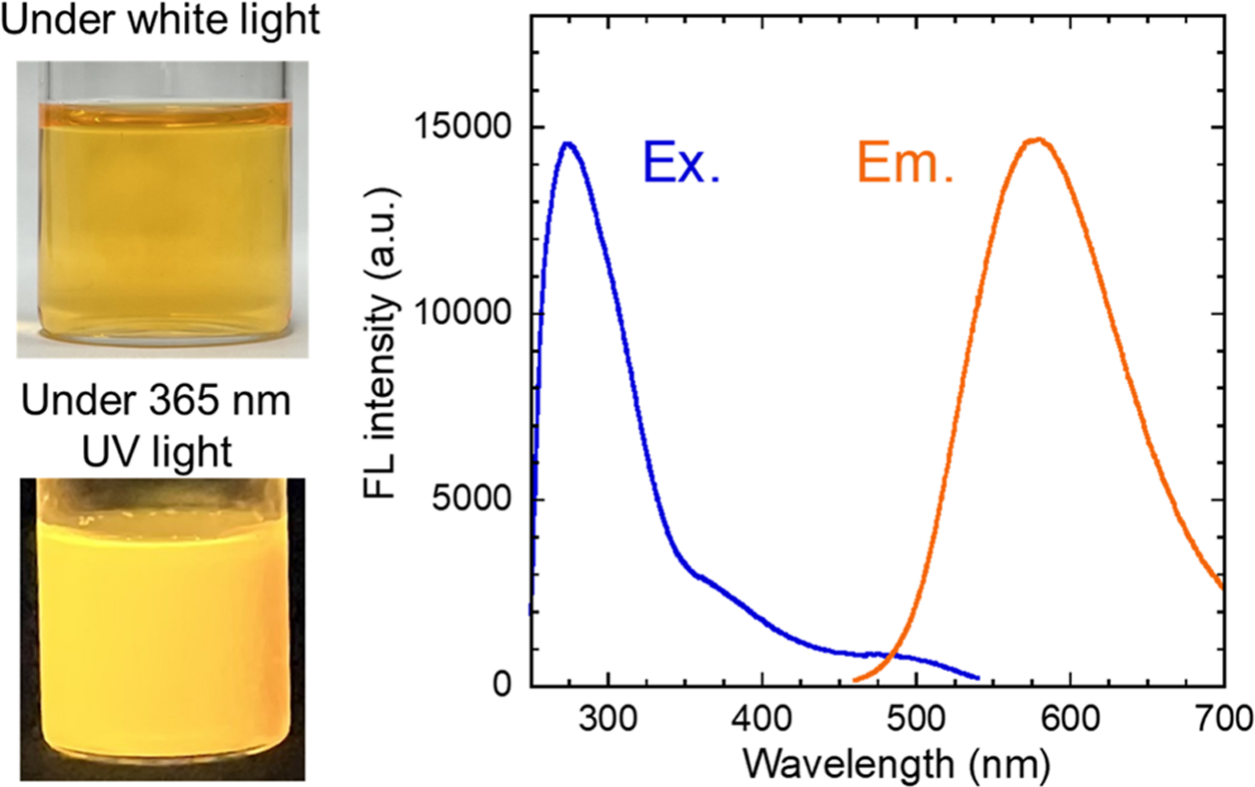
Photographs and FL excitation and emission spectra for the hydrophilized CIS/ZnS QDs dispersed in a TMAOH aqueous solution. The excitation and emission spectra were measured at the emission and excitation peak wavelengths, respectively.

UV–vis absorption spectra and Tauc plots of the hydrophobic QDs in toluene and the hydrophilized QDs in 15 wt % TMAOH solution were compared (Figure S6). The optical absorption edges in the UV–vis spectra were coincident. The band gap (*E*_g_) estimated from the Tauc plots was 2.36 eV for both samples. This is larger than 1.5 eV of bulk CIS due to the quantum size effect.^15^ FL excitation and emission spectra of the QDs dispersed in TMAOH solution are shown in Figure 3. The FL excitation and emission peaks of the QD dispersion appeared at ∼275 and ∼580 nm, respectively. It should be noted that the intensity at shorter wavelengths than ∼275 nm in the excitation spectrum was decreased by absorption of the solvent. The radiative recombination of CIS QDs has been attributed to transitions between the lower edge of the conduction band and the Cu^+^ defect levels, with some reported cases involving S^2–^ defect levels,^43^ resulting in a broad peak and a large Stokes shift. The FLQY of hydrophobic QDs in toluene was 80%, while the maximum FLQY after hydrophilization was 15% as mentioned above. Increased surface defects during the ligand-exchanging process involving desorption of surface ligands reduced the FLQY. Degradation of the QD surface could also be caused by oxidation with water;^44^ however, no FL degradation was observed for the prepared dispersion for the EPD process even after 1 week of storage, as shown in Figure S7.

### 3.3 Characterization of Fluorescent Nanocomposite Films Fabricated by EPD

**Figure 4:**
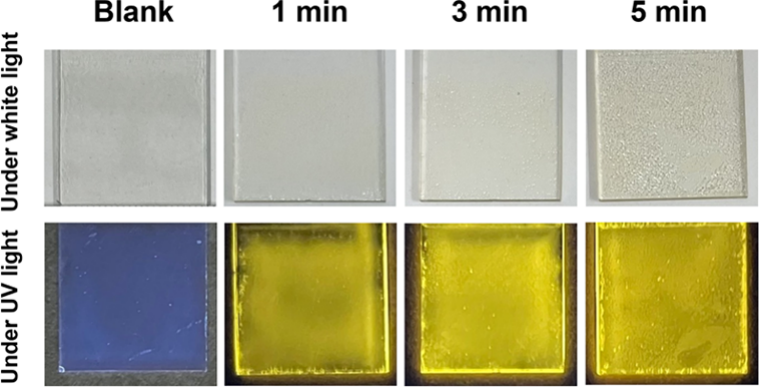
Photographs of nanocomposite films deposited for 1–5 min under white light and 365 nm UV light. Blank resin films without QDs are also shown.

Figure 4 displays the fabricated EPD films under white and 365 nm UV light. Their transparency under white light was maintained that of a blank film of silicone-modified acrylic resin without QDs. The EPD films looked uniform except for the one deposited for 5 min. The apparent yellow FL intensity of the QDs under UV light increased with increasing the deposition time. According to FL microscopy, no aggregated QDs were observed (see Figure S8). The EPD film deposited for 5 min had hemispherical holes with diameters of about 50–100 μm, indicating electrolysis of water. The theoretical electrolytic voltage of water is 1.2 V; therefore, oxygen and hydrogen were generated at the anode and cathode, respectively.^45^ The grown bubbles of oxygen gas resulted in a rough and inhomogeneous film surface.

**Figure 5:**
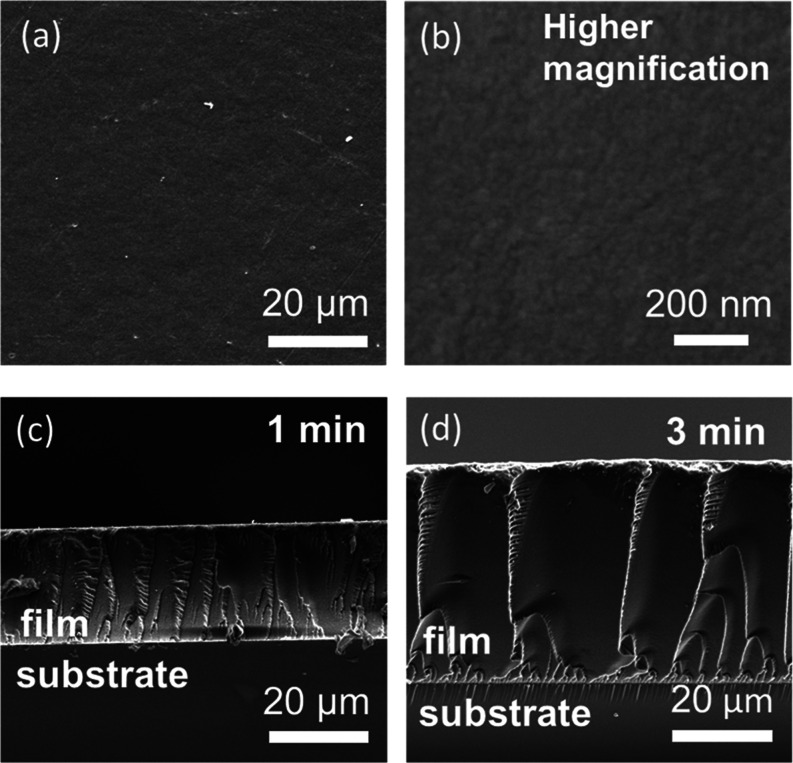
SEM images of surface and cross sections of nanocomposite films.

**Figure 6:**
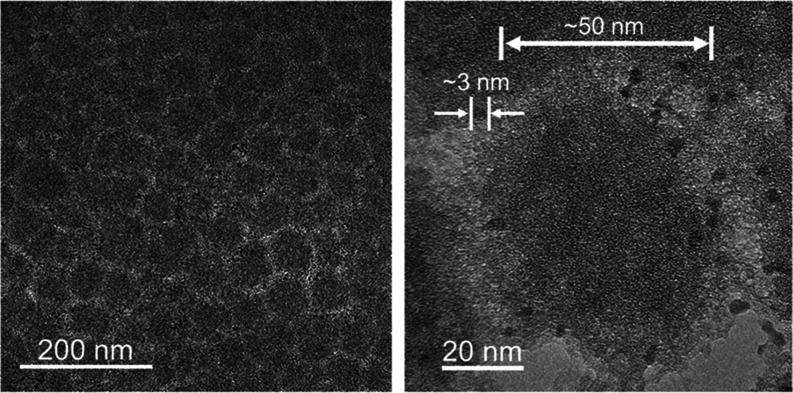
TEM images of the EPD composite film of voltage application for 30 s at lower and higher magnifications.

Figure 5 exhibits SEM images of the films deposited for 1 and 3 min. Homogeneous surfaces without holes and cracks were observed. The cross-sectional images reveal dense and homogeneous deposition on the substrate. Thicknesses in the tens of microns were achieved by deposition of the nanometer-sized materials. The film thickness measured from the cross-sectional SEM images were plotted with the deposition time in Figure S9. The thickness increased monotonically with increasing deposition time, whereas the deposition rate gradually decreased due to increased film resistance with the film growth.^46^ TEM images exhibit densely packed nanoparticles in ∼50 nm, as shown in Figure 6. Moreover, darker nanoparticles in ∼3 nm were dispersed around them. These sizes correspond to those of the silicone-modified acrylic resin and the hydrophilized QDs mentioned above (Figures S3 and S4), clearly revealing that the obtained EPD films were nanocomposites of both nanoparticles. The EPD method allows them to be deposited on an electrode directly in the liquid phase; therefore, their aggregates did not form, resulting in the uniform film structure. The number of observed QDs were much smaller than that of the resin nanoparticles in the TEM image. The QD concentration for the nanocomposite film was evaluated by comparing its elemental composition with that for the dispersion used in the EPD process. In the XRF spectrum of a nanocomposite film shown in Figure S10, S, Zn, Cu, and In derived from the QDs were detected with Si of the resin. The elemental ratio of Zn to Si in the EPD film was 4:96 regardless of the deposition time, which was close to 3:97 for the dispersion. Based on the fact that the dispersion was prepared with the QD/resin weight ratio of 3.6:96.4, the QD concentration of nanocomposite films is estimated to be ∼4 wt %. This value is consistent with the impression given by the TEM image of the nanocomposite film.

**Figure 7:**
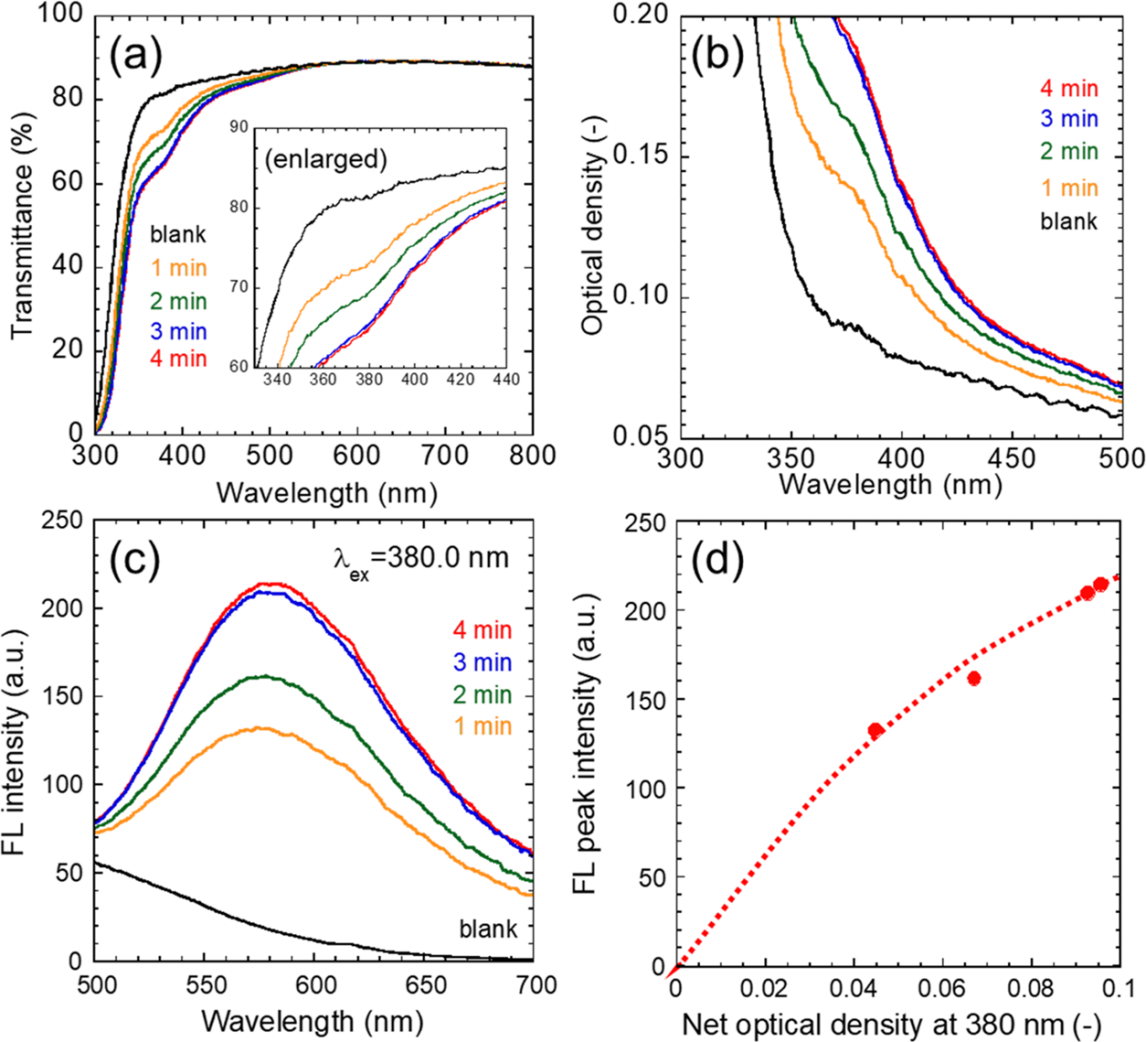
(a) Transmission spectra, (b) corresponding UV–vis optical density spectra, and (c) FL spectra of nanocomposite films deposited for 1–4 min and a blank resin film. (d) Relationship between net optical density at 380.0 nm and FL peak intensity.

As exhibited in transmission spectra of Figure 7(a), all of the nanocomposite films showed transmittance of more than 80% in the region on the longer wavelength side than 440 nm. The transmittance of nanocomposite film decreased at <440 nm with increasing thickness, while the blank resin film transmitted visible and near-UV light. Figure 7(b) shows optical density spectra converted from transmission spectra. The peak observed at <440 nm is attributed to the optical absorption of CIS/ZnS QDs. The change in this optical absorption was smaller with the longer deposition time, corresponding to the decrease in the film deposition rate. The optical density at 380 nm of each sample was subtracted from that of the blank film was calculated to obtain net optical density. The net optical density increased monotonically with increasing film thickness, as shown in Figure S11, whereas there was no proportional relationship. This violation of the Lambert–Beer law would be caused by light scattering due to microstructural inhomogeneities of the nanocomposite films. There may be tiny pores that could not be observed by the SEM observation. FL spectra of the films are compared in Figure 7(c). The FL intensity attributed to the CIS/ZnS QDs also increased monotonically with film growth. The change in FL intensity of the films under 380 nm excitation with changing the net optical density at 380 nm is plotted in Figure 7(d). The rate of increase in the FL intensity decreased with respect to the change in net optical density, which may be attributed to the effect of light scattering.

The FLQY of the nanocomposite film deposited for 3 min was 43%, which was higher than that of the basic aqueous QD dispersions shown in Table 1. Unfortunately, effective analysis could not be performed due to the low concentration of QDs in the nanocomposite film. The functional groups of the resin might passivate the QD surface, resulting in the improved FLQY. Another reason could be changes in kinetic states of the surface ligands. When the QDs were dispersed in a solvent, the surface ligands tended to move easily, and nonradiative relaxation could be likely to occur via vibrations of the surface ligands and the surrounding solvent, leading to the low FLQYs of the aqueous dispersions as shown in Table 1. In contrast, when the solvent was evaporated, the motion of the surface ligands in the dried solid film was suppressed and nonradiative relaxation could be less likely to occur. The FLQY of the obtained film was lower than ∼60% of the nanocomposite films using hydrophobic CIS/ZnS QDs in our previous work.^36^ In order to reach an FLQY comparable to those of the nanocomposite films fabricated by hydrophobic routes, the significant decrease in FLQY by the hydrophilization process must be suppressed. Optimization of the amount of MPA or the use of more appropriate hydrophilic surface ligands would improve the FLQY of nanocomposites fabricated by the aqueous route.

### 3.4 Thermal Resistance of Fluorescent Nanocomposite Films

**Figure 8:**
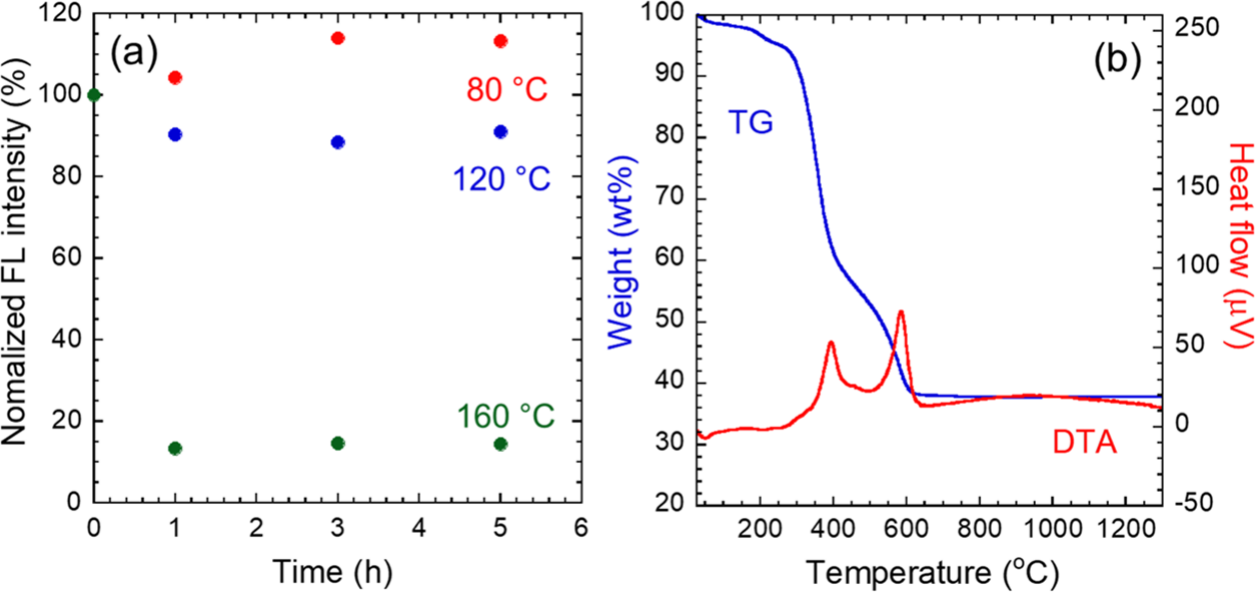
(a) Changes in FL peak intensity due to heating from the original FL spectra in Figure S12. The initial intensities are normalized. (b) TG-DTA profile of the EPD film measured under an air flow.

Fluorescent films of CIS/ZnS QDs are applicable to optoelectronic devices, including white LEDs. The temperature of LEDs rises to nearly 100 °C depending on the structure and operating power.^47^ Higher power LEDs can heat up to >150 °C during operation.^48^ Therefore, the prepared EPD films were heated at 80–160 °C for 5 h to evaluate their heat resistance. It should be noted that the sample measurements were performed after cooling to room temperature to avoid thermal quenching. There was no change in the appearance of the films after heating. The transmission spectrum of the nanocomposite film showed no change even after heating at 80–160 °C for 5 h, as exhibited in Figure S12. Figure 8(a) shows the change in the FL peak intensity at 580 nm from the FL spectrum. There was no degradation of the FL when heated to 80 °C. After heating to 120 °C, it maintained 85% of its initial intensity. These results indicate that the fabricated nanocomposites have sufficient thermal resistance when not used in high-power LEDs. On the other hand, heating at 160 °C reduced it to 14%. The maintained position of the optical absorption peak attributed to the QDs in the transmission spectra implies no change in the *E*_g_ of the QDs, which depends on the crystal structure and elemental composition of the CIS core; therefore, the FL degradation should be caused by changes in the surface state of the QDs and not the internal structure. Therefore, TG-DTA was performed to investigate the thermal degradation of the organic ligands on the QD surface. The TG-DTA results for the powdered nanocomposite film are shown in Figure 8(b). A small endothermic peak is observed below 100 °C, which is attributed to the vaporization of water molecules incorporated into the EPD film. Weight loss with exotherm was observed at ∼400 and ∼590 °C. Based on the TG-DTA profiles of the hydrophilic QDs and resin (see Figure S13), they are attributed to combustion of organic ligands such as MPA and acrylic chains of the resin. The slight weight loss due to water desorption only was observed below 200 °C, indicating that the thermal degradation of the nanocomposite film heated at 160 °C was not caused by thermal damage to the organic materials. From these results, the FL degradation may be caused by the formation of surface defects accompanied by thermally induced desorption of surface ligands on the QDs. The formed surface defects would increase the nonradiative relaxation probability, resulting in the decreased FL intensity.

### 3.5 Potential Applications of the EPD Films

**Figure 9:**
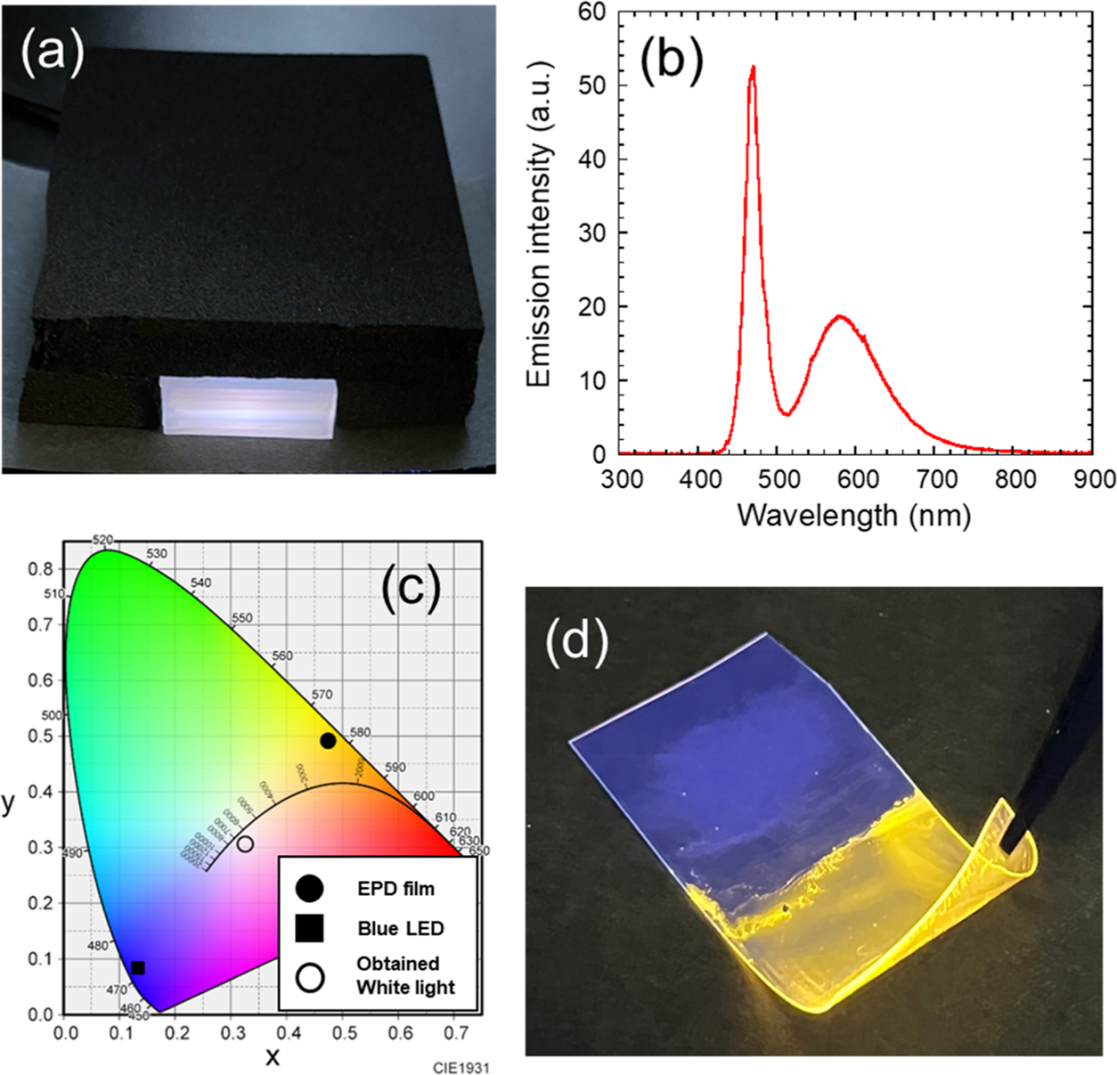
(a) Demonstration of a white LED device using the EPD film on a commercial flat-panel blue LED. (b) Emission spectrum of the device and (c) corresponding chromaticity coordinate. Chromaticity coordinates of individual emissions for the used EPD film and blue LED are also shown. (d) Demonstration of flexibility of the EPD film fabricated on an ITO-coated PET substrate.

We demonstrated a white LED device by combining the resulting EPD films with a blue LED. As displayed in Figure 9(a), white luminescence was observed. It should be noted that the power of the blue LED was adjusted to obtain the white light. Its emission spectrum shown in Figure 9(b) exhibited a sharp emission peak of the blue LED and a broad FL peak of the CIS/ZnS QDs at 470 and 581 nm, respectively. The chromaticity coordinates converted from this spectrum were (0.326, 0.306), which were located between the emission colors of the blue LED and the EPD film, as plotted in Figure 9(c). White light was successfully realized by the EPD film containing CIS/ZnS QDs. Furthermore, the silicone-modified acrylic resin provides good flexibility for the obtained EPD film. The resulting nanocomposite film is highly adherent and difficult to peel off from the substrate, whereas the EPD film can be fabricated on various conductive materials. Figure 9(d) demonstrates the high flexibility of an EPD film obtained on an ITO-coated poly(ethylene terephthalate) (PET) substrate, indicating possible contributions to thin-film optoelectronic devices.

## 4 Conclusions

In this work, hydrophilic CIS/ZnS QDs were prepared by adsorbing MPA as a surface ligand to fabricate fluorescent nanocomposite films by aqueous EPD with silicone-modified acrylic resin nanoparticles. Both negatively charged nanoparticles were simultaneously dispersed in water at pH 10.0 and deposited on the transparent conductive substrate at the anode side by applying 4 V for 1–5 min. The thickness of the nanocomposite film increased with an increase in the voltage application time. A lot of bubble traces were observed in the EPD film after 5 min of deposition, which was attributed to the oxygen gas generated on the anode by the electrolysis of water. SEM observation showed that the EPD films were uniformly and densely deposited on the substrates. Further microscopic observation by TEM revealed that the QDs were dispersed around the resin nanoparticles. These results indicated that both nanoparticles were deposited from the dispersion without significant aggregation. The nanocomposite films showed high transmittance of >80% in the visible region. The optical absorption peak of the CIS/ZnS QDs appeared at ∼440 nm. The optical density at 380 nm increased with the growth of the nanocomposite film. The absolute FLQY was 43%. The FL intensity increased with increasing absorption for 380 nm excitation light; however, no linear relationship was found between them, possibly due to light scattering caused by the inhomogeneous microstructure. When the nanocomposite films were heated at 120 °C for 5 h to evaluate the thermal stability, the FL intensity maintained 85% of the initial value, while it decreased to 14% at 160 °C. No thermal degradation was observed under heating at 80 °C, indicating that the nanocomposite films are durable against heating in LED devices up to ∼100 °C. A white LED device was demonstrated by combining a blue LED and the fabricated nanocomposite film. The observed emission color was white with chromaticity coordinates of (0.326, 0.306). Furthermore, when EPD was performed using an ITO-coated PET substrate, the resulting nanocomposite film showed high flexibility. These demonstrations indicate that the fabricated fluorescent nanocomposite films are promising for various optoelectronic applications such as white LEDs and flexible devices. If other fluorescent QDs are successfully hydrophilized and used instead of the CIS/ZnS QDs, then the aqueous EPD technique of this work will broaden the scope of applications.

## Supporting Information Available

FL measurement method for an EPD film sample using an integrating sphere (Figure S1); XRD profiles of hydrophobic CIS/ZnS QDs and hydrophilized QDs (Figure S2); TEM images and corresponding size distributions of hydrophobic and hydrophilized QDs (Figure S3); TEM image of silicon-modified acrylic resin nanoparticles and corresponding size distribution (Figure S4); photographs of hydrophilized CIS/ZnS QDs dispersed in aqueous TMAS, TMAOH, and NaOH solutions at different pH values (Figure S5); UV–vis absorption spectra and corresponding Tauc plots for the hydrophobic QDs in toluene and hydrophilized QDs in TMAOH solution (Figure S6); change in FL spectrum of the dispersion for EPD after preparation (Figure S7); bright field and fluorescent microscope images of nanocomposite films deposited for 1–5 min (Figure S8); change in film thickness with deposition time of the EPD process (Figure S9); XRF spectra of powdered samples (Figure S10); change in net optical density at 380 nm of nanocomposite film with thickness (Figure S11); changes in transmission spectra and FL spectra due to heating (Figure S12); TG-DTA profiles of dried hydrophilized QDs and resin nanoparticles under air flow (Figure S13) (PDF)
