# Supplementary material for: Eco-Friendly Electrophoretic Deposition of Fluorescent Nanocomposite Films in an Aqueous Dispersion of Hydrophilized Core/Shell CuInS2/ZnS Quantum Dots for Optoelectronic Applications
Source: ACS Appl Mater Interfaces. 2024 Feb 5;16(6):7780–9. doi: 10.1021/acsami.3c17264 (PMC10876053; doi:10.1021/acsami.3c17264)
Supplement: Supplementary file 1 — am3c17264_si_001.pdf [file am3c17264_si_001.pdf]

Supporting Information

ACS Applied Materials & Interfaces

Eco-Friendly Electrophoretic Deposition of Fluorescent Nanocomposite Films in an Aqueous Dispersion of Hydrophilized Core/Shell $CuInS_2$/ZnS Quantum Dots for Optoelectronic Applications

Asshu Morimoto, Yoshiki Iso,* and Tetsuhiko Isobe*

Department of Applied Chemistry, Faculty of Science and Technology, Keio University, 3-14-1 Hiyoshi, Kohoku-ku, Yokohama 223-8522, Japan

*Corresponding Authors:

Yoshiki Iso – E-mail: iso@applc.keio.ac.jp; Tel.: +81 45 566 1558; Fax: +81 45 566 1551; orcid.org/0000-0001-7483-2828

Tetsuhiko Isobe – E-mail: isobe@applc.keio.ac.jp; Tel.: +81 45 566 1554; Fax: +81 45 566 1551; orcid.org/0000-0002-0868-5425

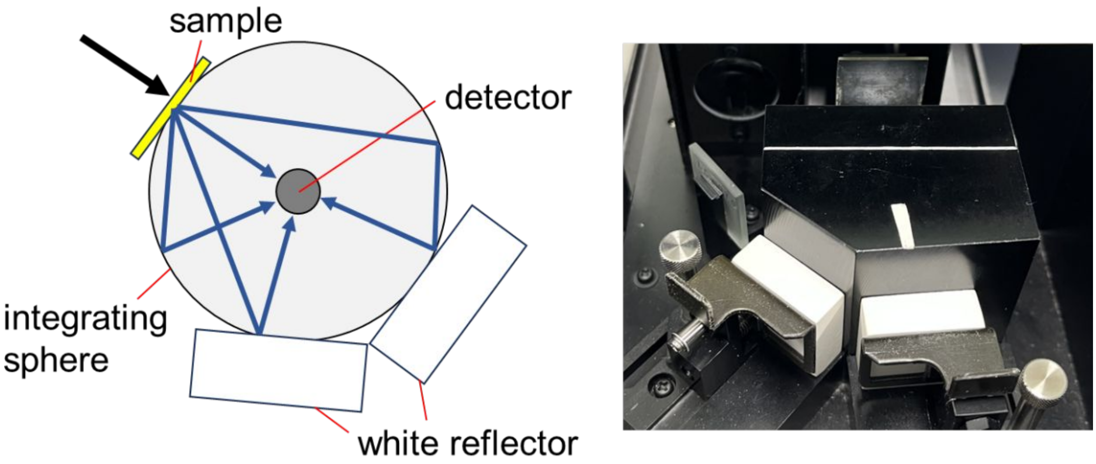

Figure S1. Schematic illustration and photograph of PL measurement for an EPD film sample using an integrating sphere.

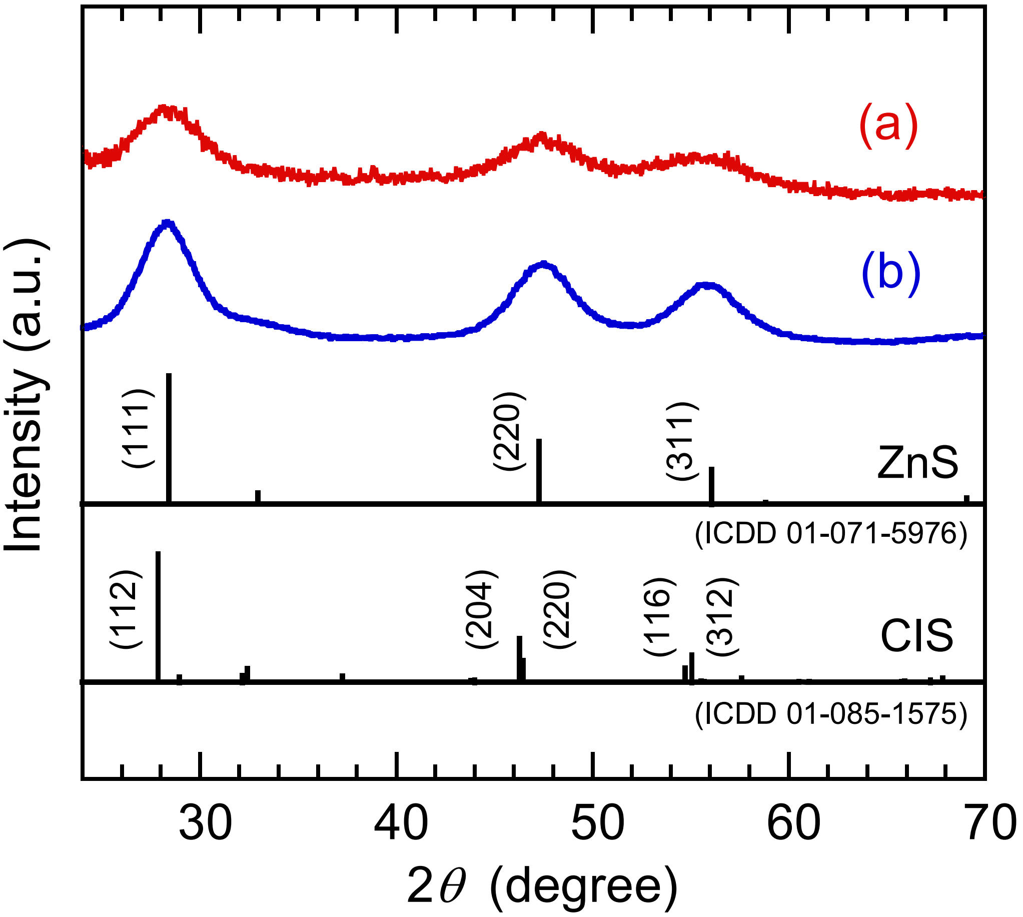

Figure S2. XRD profiles of (a) hydrophobic CIS/ZnS QDs and (b) hydrophilized QDs.

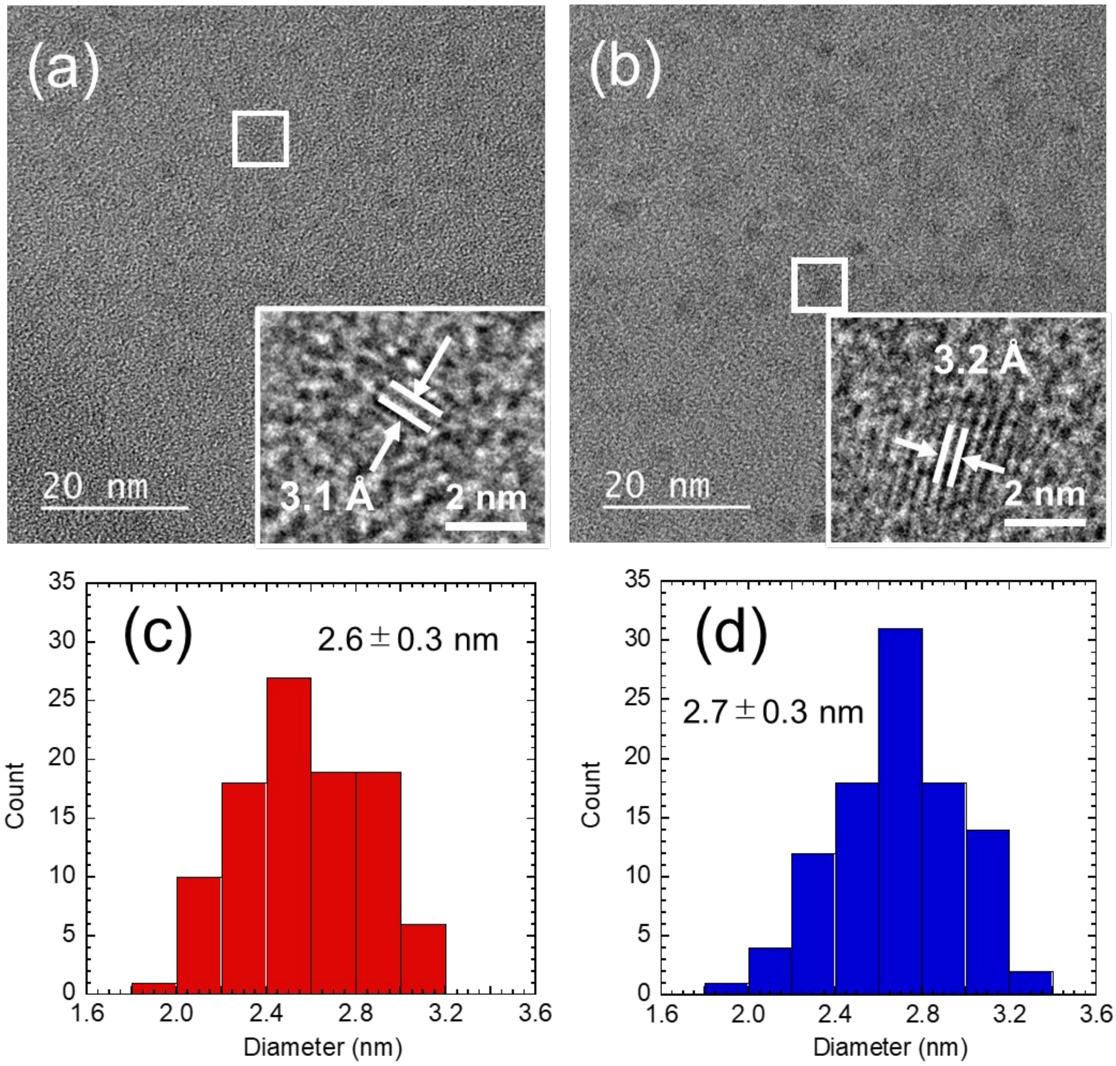

Figure S3. (a,b) TEM images and (c,d) corresponding size distributions of (a,c) hydrophobic and (b,d) hydrophilized QDs.

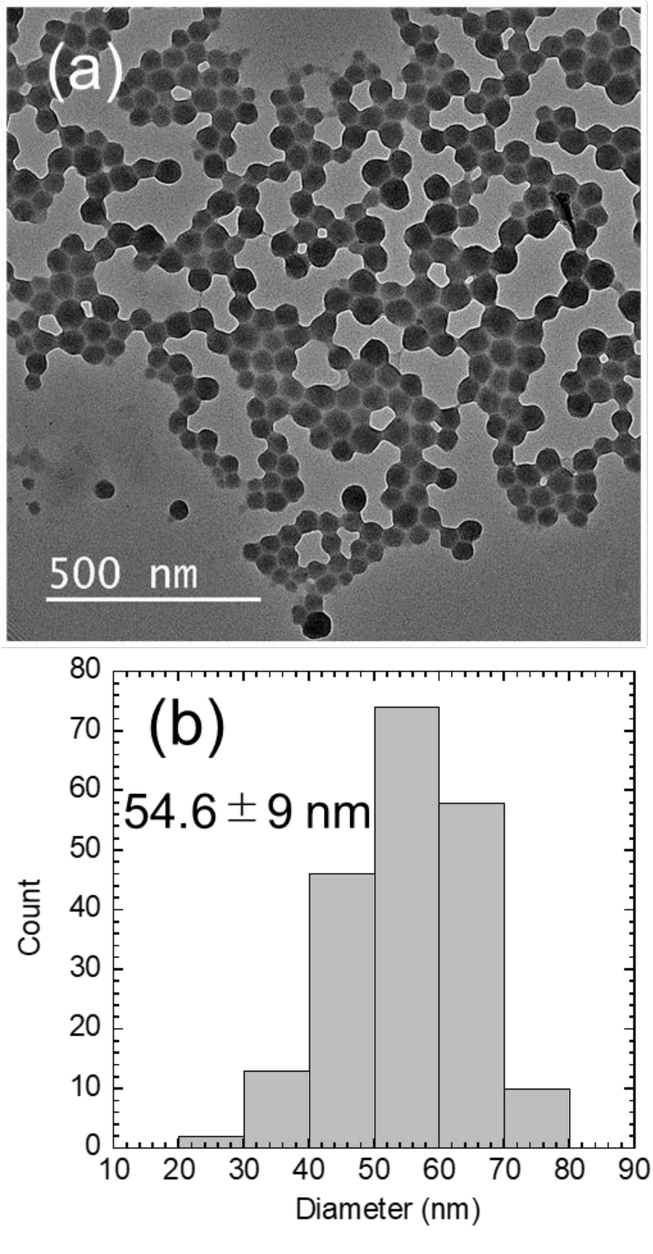

Figure S4. (a) TEM image and (b) corresponding size distribution of silicon-modified acrylic resin nanoparticle.

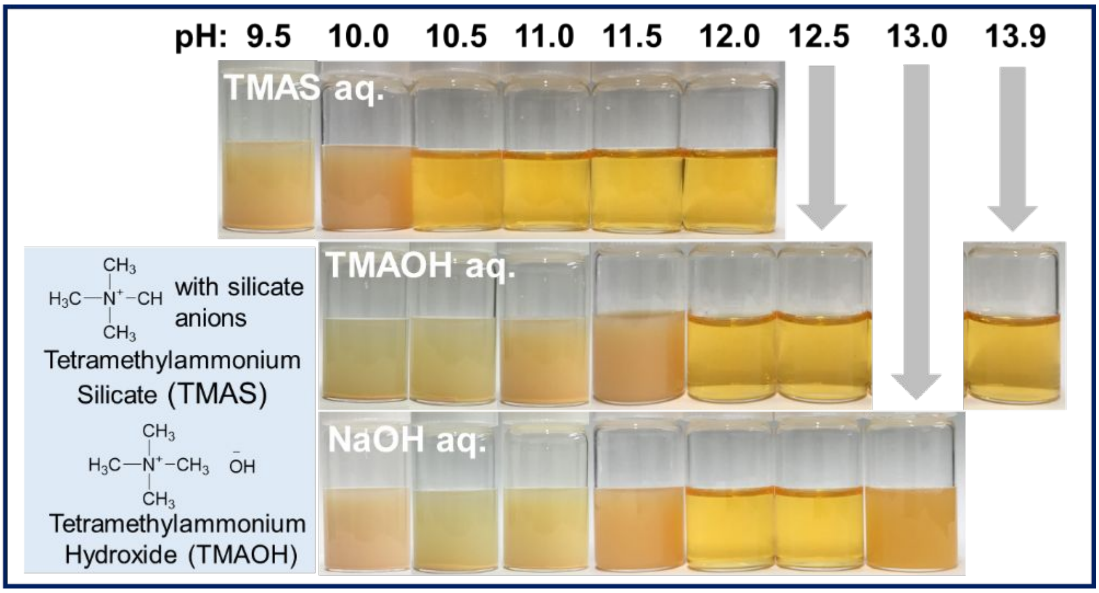

Figure S5. Photographs of hydrophilized CIS/ZnS QDs dispersed in aqueous TMAS, TMAOH and NaOH solutions. The pH values of the as-purchased TMAS and TMAOH solutions were 12.0 and 13.9, respectively. They were diluted with ultrapure water to adjust the pH. 30 mg of QDs was added to 10 mL of the respective dispersant with adjusted pH. The concentration of QDs is 3 g L^{-1}.

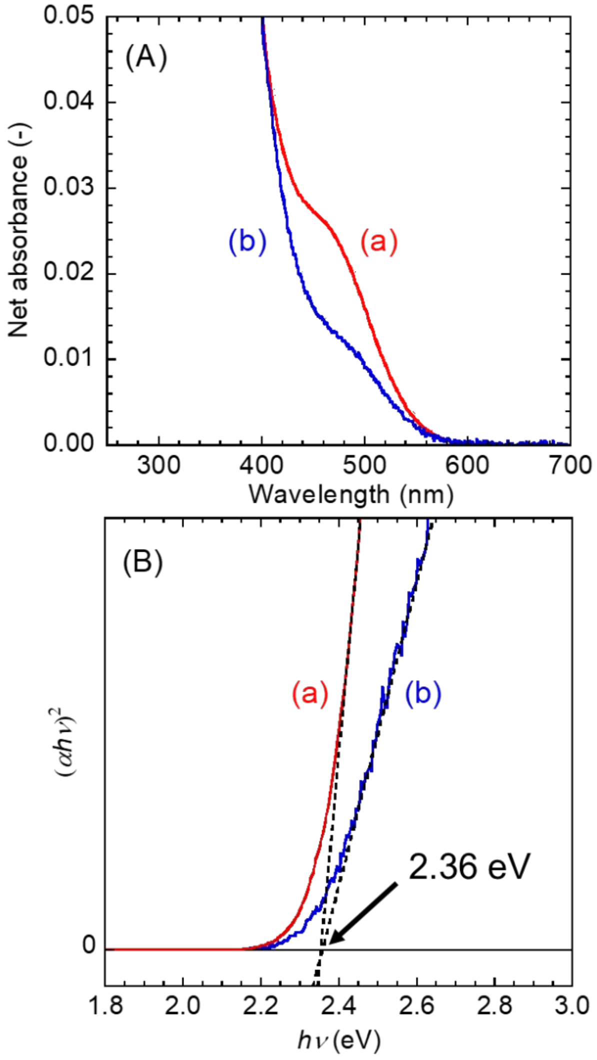

Figure S6. (A) UV-vis absorption spectra and (B) corresponding Tauc plots for the (a) hydrophobic QDs in toluene and (b) hydrophilized QDs in 15 wt% TMAOH aqueous solution. Their net absorbance at 400 nm was adjusted to 0.05 by controlling the QD concentration. The net absorbance of the dispersed QDs was calculated by subtracting the absorbance of each solvent as a blank.

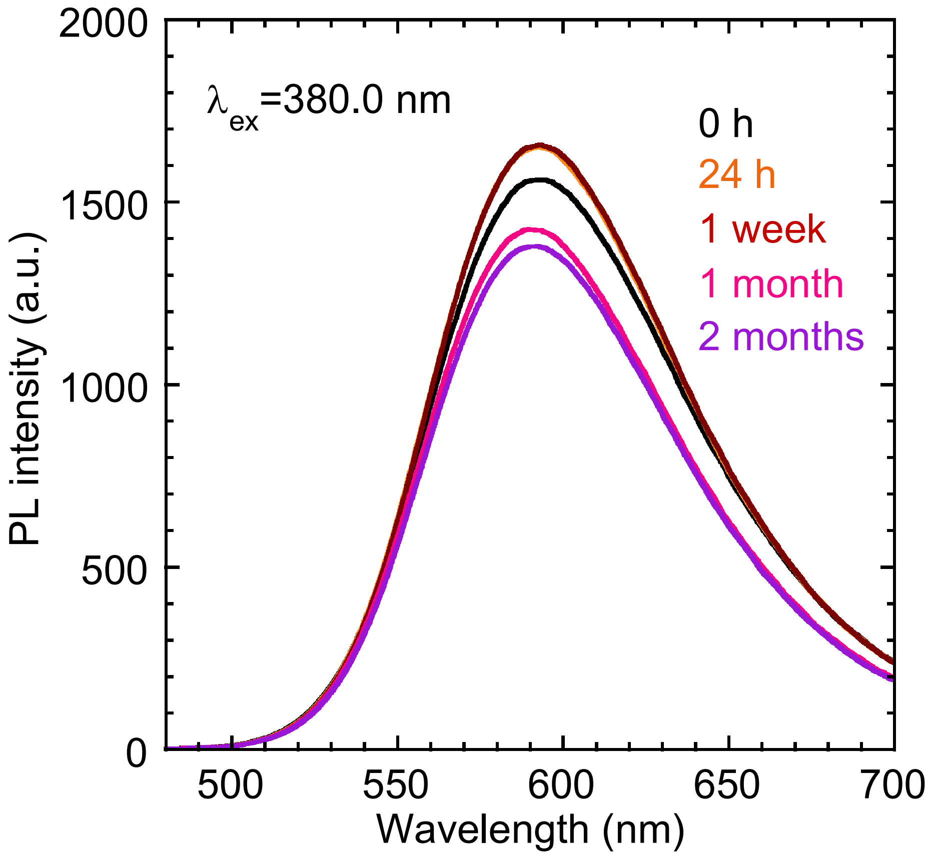

Figure S7. Change in FL spectrum of the dispersion for EPD after preparation. The FL intensity did not decrease until 1 week, indicating that the hydrophilized QDs were not immediately degraded by water. The slight FL increase observed by 24 h could not be clarified. This might be caused by photoactivation under the excitation light during the measurement.[S1] The decrease of fluorescence intensity observed afterward might indicate a slow progress of QD degradation by water. The dispersion maintained higher FL intensity even after 2 months. Sediment was not observed during the experiment.

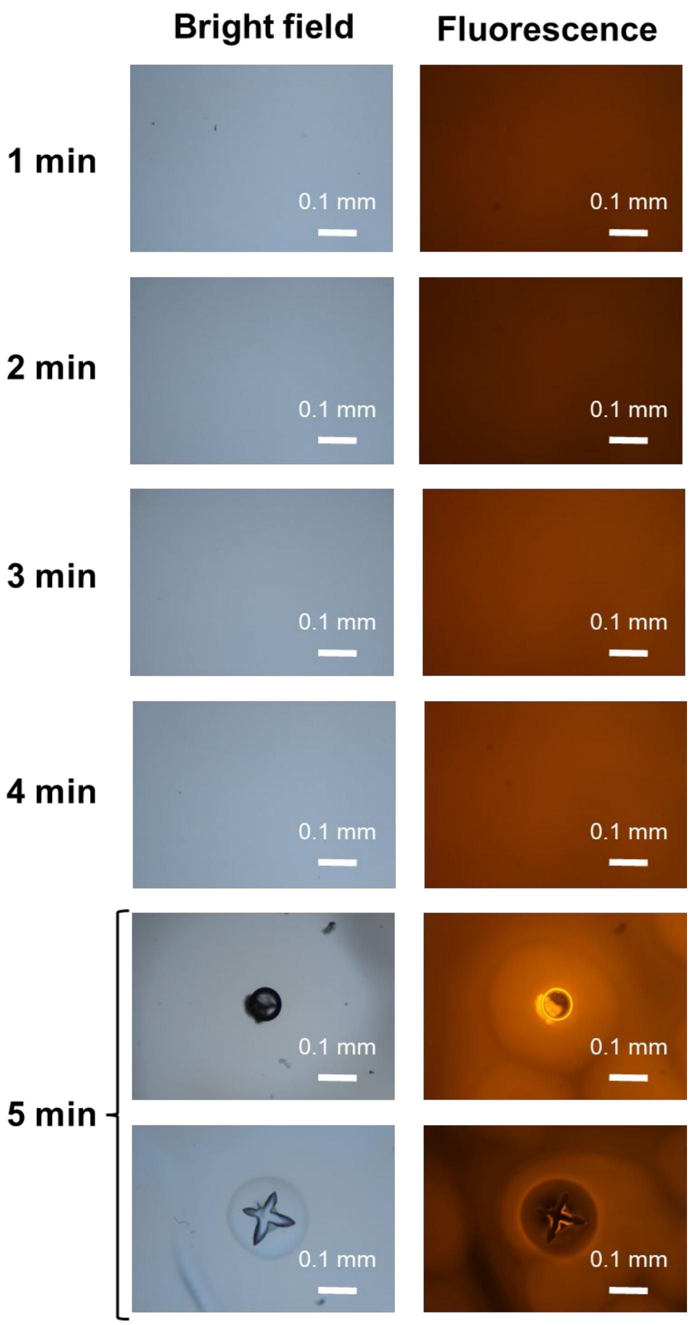

Figure S8. Bright field and fluorescence microscope images of nanocomposite films deposited for 1–5 min.

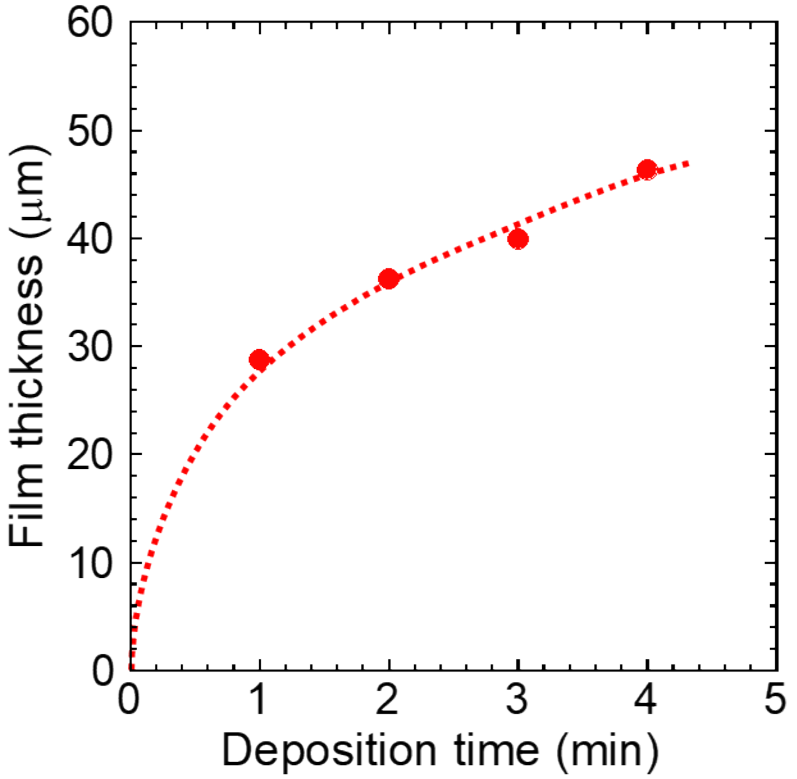

Figure S9. Change in thickness of nanocomposite film with deposition time of the EPD process.

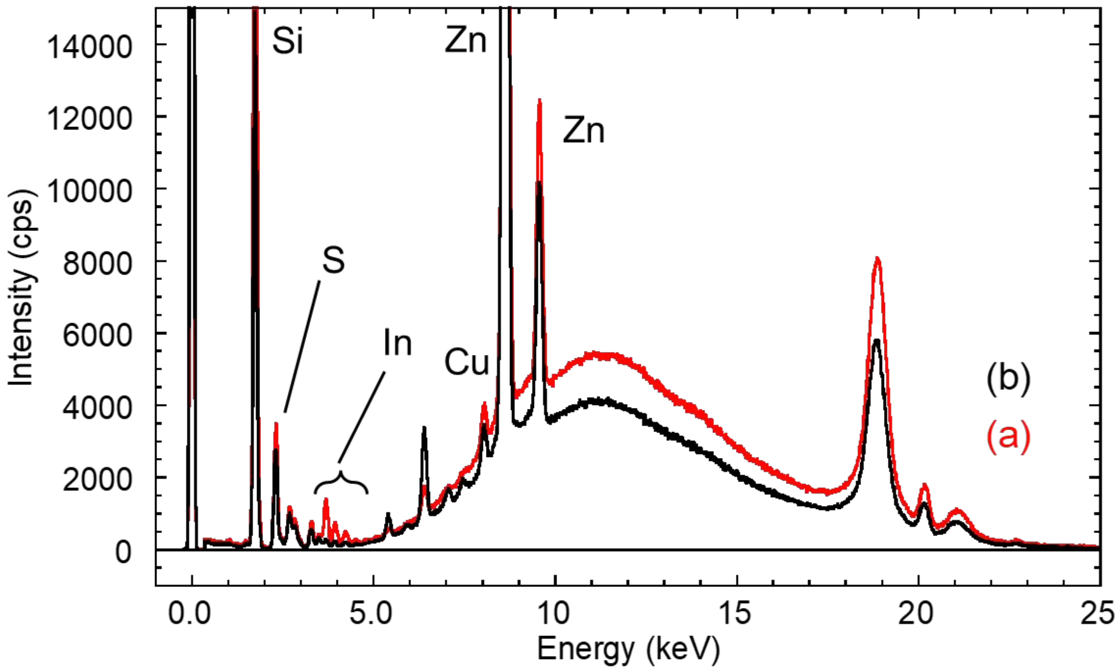

Figure S10. XRF spectra of powdered samples. (a) nanocomposite film deposited for 4 min and (b) dried dispersion used in the EPD process. The nanocomposite film was collected from the substrate before drying to avoid detecting In of the ITO coating.

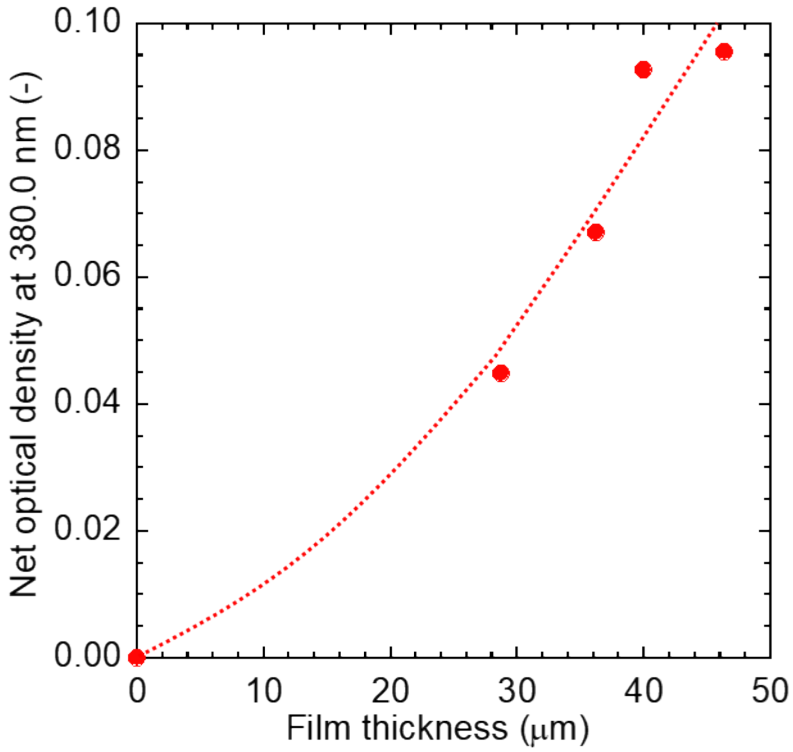

Figure S11. Change in net optical density at 380 nm of nanocomposite film with thickness.

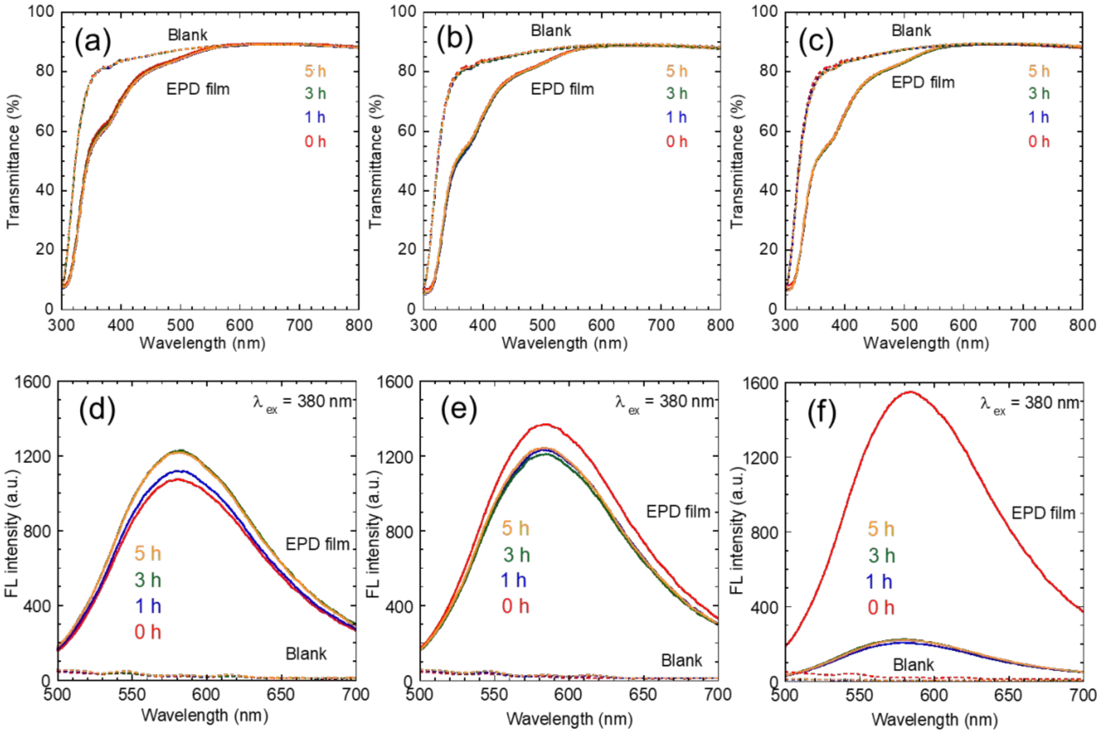

Figure S12. Changes in (a–c) transmission spectra and (d–f) FL spectra due to heating.

The heating temperatures are (a,d) 80 °C, (b,e) 120 °C, and (c,f) 160 °C.

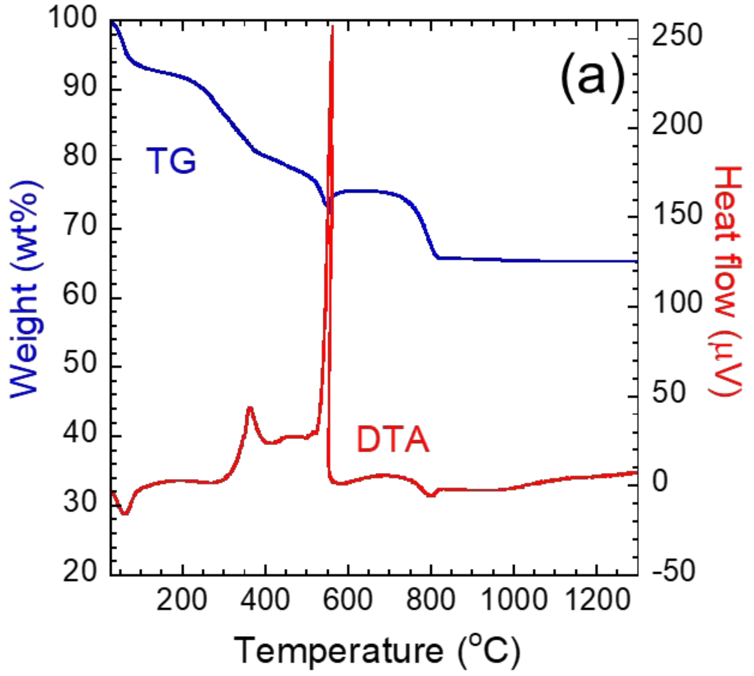

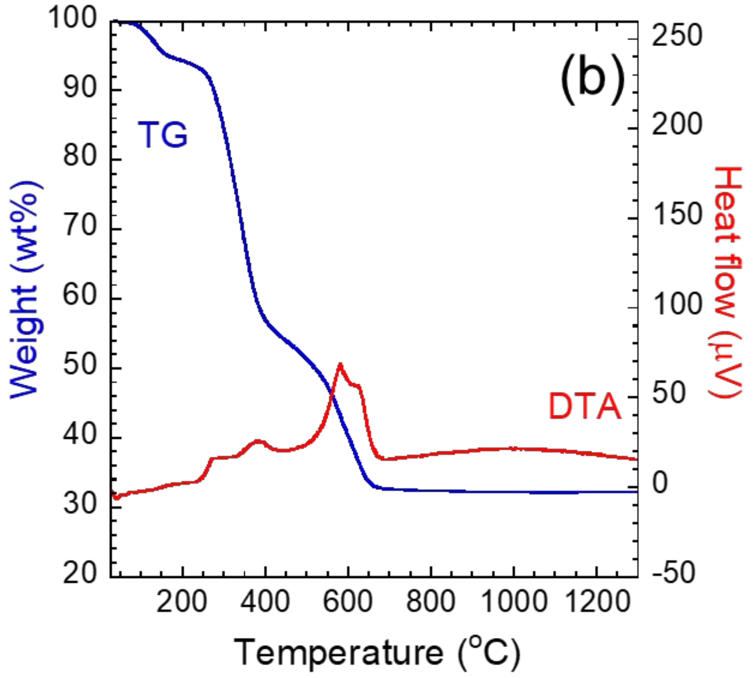

Figure S13. TG-DTA profiles of dried (a) hydrophilized QDs and (b) resin nanoparticles under air flow.

REFERENCE

S1) Wada, C.; Iso, Y.; Isobe, T.; Sasaki, H. Preparation and Photoluminescence Properties of Yellow–Emitting $CuInS_2/ZnS$ Quantum Dots Embedded in TMAS–Derived Silica. *RSC Adv.* **2017**, *7*, 7936–7943.
